# *In vitro* Models of Breast Cancer Metastatic Dormancy

**DOI:** 10.3389/fcell.2020.00037

**Published:** 2020-03-03

**Authors:** Marco Montagner, Erik Sahai

**Affiliations:** ^1^Department of Molecular Medicine, School of Medicine and Surgery, University of Padua, Padua, Italy; ^2^The Francis Crick Institute, London, United Kingdom

**Keywords:** cancer dormancy, metastatic dormancy, *in vitro* models cancer, cancer metastasis, breast cancer, metastasis biology

## Abstract

Delayed relapses at distant sites are a common clinical observation for certain types of cancers after removal of primary tumor, such as breast and prostate cancer. This evidence has been explained by postulating a long period during which disseminated cancer cells (DCCs) survive in a foreign environment without developing into overt metastasis. Because of the asymptomatic nature of this phenomenon, isolation, and analysis of disseminated dormant cancer cells from clinically disease-free patients is ethically and technically highly problematic and currently these data are largely limited to the bone marrow. That said, detecting, profiling and treating indolent metastatic lesions before the onset of relapse is the imperative. To overcome this major limitation many laboratories developed *in vitro* models of the metastatic niche for different organs and different types of cancers. In this review we focus specifically on *in vitro* models designed to study metastatic dormancy of breast cancer cells (BCCs). We provide an overview of the BCCs employed in the different organotypic systems and address the components of the metastatic microenvironment that have been shown to impact on the dormant phenotype: tissue architecture, stromal cells, biochemical environment, oxygen levels, cell density. A brief description of the organ-specific *in vitro* models for bone, liver, and lung is provided. Finally, we discuss the strategies employed so far for the validation of the different systems.

## Metastatic Dormancy

Dormancy is an old concept that describes a clinical phenomenon (Klein, 2011; Uhr and Pantel, 2011), i.e., the relapse of a cancer after surgical removal of the primary tumor in a patient considered clinically disease-free for a long time. This implies that cancer cells disseminated prior to surgery and persisted as Minimal Residual Disease (MRD) for a prolonged time (arbitrarily defined, but usually longer than 5 years) before switching to aggressive growth and overt metastasis. The recurrence can be at the primary site (primary tumor dormancy) or at a secondary site (metastatic dormancy). The mechanisms underlying the two types of dormancy are likely to be partially overlapping if involving cell intrinsic genetic/epigenetic mechanisms, or distinct, if dependent on the tissue microenvironment. Clinical dormancy is common for breast, prostate, melanoma, renal, and thyroid cancers, while it is rarely observed in lung and colon cancers (Uhr and Pantel, 2011). In breast cancers, estrogen receptor (ER) status seems to profoundly influence the rate of relapse: ER− patients tend to recur within the first 5 years following primary tumor diagnosis, while ER+ patients have increased risk between 5 and 20 years (Pan et al., 2017; Pantel and Hayes, 2018). While anti-estrogen therapy significantly improved patient outcomes, a significant fraction of them still develops distant relapses and extending the duration of the treatment beyond 5 years yields little benefit (Pan et al., 2017; Bense et al., 2018; Pantel and Hayes, 2018).

In this review we specifically focus on *in vitro* models developed to study metastatic dormancy. Upon dissemination in a secondary organ, metastatic breast cancer cells (BCCs) can undergo three fates: death, dormancy, or growth. Dormancy does not have a clear biological definition, it has been proposed a classification of dormant phenotypes into cellular dormancy (entering into reversible quiescence in G0) and tumor mass dormancy (a small cluster of cells where proliferation is counterbalanced by apoptosis due to lack of nutrients, blood supply or because of immune surveillance) (Linde et al., 2016; Goddard et al., 2018; Weidenfeld and Barkan, 2018; Lan et al., 2019). However, these states are likely to coexist within the same patients and probably the same cells can dynamically fluctuate between these different states.

Growth arrest mechanisms generally fall into three main categories: quiescence, terminal differentiation and senescence (Pack et al., 2019). While the former is reversible upon withdrawal of restrictive signals, the latter is associated with permanent exit from cell cycle and persistent activation of stress signals. Cyclin-dependent kinases (CDKs) coupled with cyclins promote cellular proliferation by inhibiting pocket protein family (Rb, p107, p130), conversely CDK-cyclin couples are inhibited by CIP/KIP inhibitors (p21, p27, p57) and INK4 inhibitors (like p16). Intrinsic and extrinsic factors are integrated into the regulation of this core machinery, for example, serum starvation triggers upregulation of p27 and exit from proliferation, while CDKs are induced by mitogenic signals. DNA damage is the strongest internal signal regulating proliferation and mediates growth arrest via stabilization of p53 and its target p21. Apart from the prominent role of p27 (Bragado et al., 2013; Touny et al., 2014), little is known about the role of cell-cycle machinery in the different stages of metastatic dormant phenotype and whether dormant cells lie closer to quiescence or senescence in the growth arrest spectrum.

Several strategies have been implemented to visualize dormant disseminated cells *in vivo*. The easiest methods are staining of fixed tissues for the proliferation-associated protein Ki67, growth arrest marker p27 or for DNA-incorporated synthetic nucleosides (such as BrdU or EdU) (Ghajar et al., 2013; Fluegen et al., 2017; Carlson et al., 2019; Montagner et al., 2020). The main limit of these methods is that they are not compatible with tissue viability and don’t allow isolation of non-proliferating cells. To circumvent this problem, De Cock et al. (2016) utilized an intracellular fluorescent vital dye to label cells prior to injection into mice. The dye is diluted at each cell division, allowing for isolation of cells that didn’t proliferate (Cock et al., 2016). Similarly, Fluegen et al. (2017) generated metastatic cells stably expressing a photoconvertible fluorescent protein, Histone 2B-Dendra2. This is photoconverted from green to red before injecting cells in mice, and nuclear red fluorescent signal decreases at each cell division, similar to a vital dye (Fluegen et al., 2017). The fluorescence ubiquitination–based cell cycle indicator (FUCCI) system has also been applied (Albrengues et al., 2018) which allows dynamic visualization of each phase of the cell cycle during *in vivo* imaging.

Whether dormant cells are quiescent or undergo a balanced proliferation (where proliferation rate is compensated by apoptosis) has a profound impact on the design of new therapies (Wells et al., 2013), because it is assumed that dormant cells are inherently resistant to conventional chemotherapy as they are not cycling (Wells et al., 2013; Linde et al., 2016). This is not entirely correct as recent data show that chemoresistance is in part actively supported by the metastatic niche and is not just a consequence of cell-cycle arrest (Carlson et al., 2019). Moreover, it has been recently shown that several patients with bone marrow-disseminated cancer cells (DCCs) that resisted treatment with FEC (fluorouracil, epirubicin, and cyclophosphamide), benefited from additional treatment with docetaxel; as this drug induces microtubule stabilization, cell-cycle arrest in the G(2)M phase and apoptosis, this suggests that a considerable fraction of dormant cells still has proliferative activity (Naume et al., 2014; Goddard et al., 2018). Notably, patients with dormant DCCs that persisted after the second therapy had worst prognosis, further supporting the idea that metastatic lesions develop from pre-existing dormant DCCs (Braun et al., 2005; Naume et al., 2014).

## Data From Patients

Despite the fact that metastatic lesions account for the vast majority of cancer-related deaths, metastatic colonization is an extremely inefficient process (Massagué and Obenauf, 2016). Each step of the hematogenous metastatic cascade of epithelial cancers (loss of polarity, detachment from primary tumor, migration through basal membrane and stromal layers, intravasation, survival in the blood stream, extravasation) represents a significant hurdle that contributes to the selection of aggressive cancer cell clones. Even focusing on the steps that follow intravasation, less than 0.01% of cells will eventually develop metastatic lesions and not even in all patients (Naumov et al., 2002; Braun et al., 2005). These numbers are confirmed by experimental models of metastatic dissemination (Valastyan and Weinberg, 2011), with the switch from micrometastasis to macrometastasis estimated to happen with a frequency lower than 0.02% for liver metastases from melanoma cells (Luzzi et al., 1998; Cameron et al., 2000). From these numbers the expectation might be that persistence in secondary organs is a feature restricted to few highly aggressive cells (seed) and/or to target organs with a peculiar permissive environment (soil). Yet, clinical and experimental evidence of early dissemination of breast cancers have been reported (Goddard et al., 2018), indicating that even cells from early stage disease can disseminate and persist. Moreover, several registries reported people who have developed cancers following organ transplants (Buell et al., 2005; Klein, 2011), indicating that disseminated cells survived in a quiescent state in different organs of donors with prior undiagnosed or cured cancers. Of note, transplanted organs were not the most common sites of metastasis, such as kidney or heart. This evidence supports the idea that survival after metastatic spreading might not be limited, *per se*, to highly aggressive cells or few target organs, and that indolent disease can seed additional sites.

## Challenges for the Development of *In Vitro* Models

Transgenic mouse models of dormant/indolent metastatic mammary cancers have been described over the years (Li et al., 2000; Hüsemann et al., 2008) and have been recently used to discover the roles of progesterone receptor, Her2 and partial-EMT into early dissemination (Harper et al., 2016; Hosseini et al., 2016). However, these models also have significant limitations, such as the hurdles associated with tracking asynchronous disseminated metastatic cells. Moreover, dormancy is often the result of the crosstalk between the cancer cells and the metastatic stroma; thus, parameters should be modulated at single cell resolution, which is often impossible *in vivo*. Lastly, removing single stromal populations *in vivo* to prove their requirement into control of dormancy is incompatible with animal viability; the design of *in vitro* models is a valuable strategy to bypass these limitations.

**FIGURE 1:**
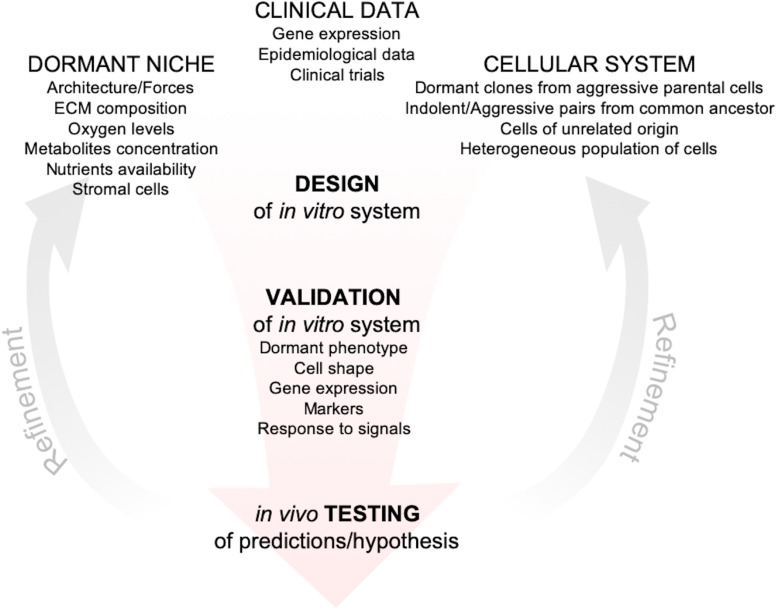
Framework for the development of *in vitro* models of metastatic dormancy. *In vitro* models should include one or more features of the dormant niche starting from measurements/observations of the target metastatic tissue *in vivo*. Then cellular systems have to be chosen based on clinical evidences that support their specific use, i.e., correct metastatic tropism, correct gene expression (when available), correct response to signals (such as inflammation). After the initial setup, the *in vitro* system must be validated and the cell lines, as well as the microenvironment, should exhibit one or more features specific of the dormant phenotype (obtained from animal models or clinical data). The *in vitro* system should then be exploited to generate hypothesis and prediction that could be tested experimentally in animal models or from clinical data. Finally, according to the feedbacks from the *in vivo* validation, the system can be refined for more accurate predictions and hypothesis.

The development of reliable *in vitro* models to investigate dormancy is hampered by the limited data from patients (Chéry et al., 2014; Vishnoi et al., 2015). Scattered dormant DCCs lie far below the radar of current diagnostic tools and significant advancements in that direction will be challenging and will run the risk of detecting lesions that would never progress (Srivastava et al., 2019). Thus, together with new tools for detection of metastatic clusters at single-cell resolution, development of markers for dangerous vs. harmless disseminated cells are highly desirable. Over the last decade, in parallel with advances in microfluidic technologies, biomaterials and biofabrication techniques, many groups developed and optimized *in vitro* tools to explore the issue of metastatic dormancy with different objectives, from discovery of basic mechanisms of survival to platforms for high-throughput drug discovery (Pradhan et al., 2018; Rao et al., 2019). Even though these *in vitro* models are increasing in number and complexity, their descriptive and/or predictive power is unknown, given the paucity of markers, metrics and expression data from patients. Nevertheless, there are common themes emerging from different models that led to the approval of clinical trials (Goddard et al., 2018) and to the development of tools to predict likelihood of relapse (Borgen et al., 2018). Moreover, recent publications provided explanations for epidemiological data linking inflammation with higher risk of breast cancer relapse (Cock et al., 2016; Albrengues et al., 2018). Recent reviews have covered in depth the history, evolution and recent advances in the dormancy field (Giancotti, 2013; Ghajar, 2015; Linde et al., 2016; Aguirre-Ghiso and Sosa, 2018; Goddard et al., 2018), this review focuses instead on *in vitro* models for breast cancer metastatic dormancy that have been more extensively validated and that, regardless of their complexity, led to discoveries supported by independent *in vitro* systems, animal models or by data from patients. Moreover, we provide a framework for the development of further *in vitro* models, by critically discussing metrics and parameters that should ideally be integrated to tightly anchor new and old models with data from animal models or breast cancer patients with the hope of circumventing the limitations discussed above (Figure 1).

## Cells

To establish *in vitro* models that reflect the *in vivo* situation, it is first necessary to have cells that exhibit dormant behavior *in vivo*, several BCCs lines with different dormant proclivity and tropism have been generated.

The first option is the use of cell lines derived from *in vivo* selection of dormant clones from an aggressive parental cell line (comparison between parental and selected subclones). A list of those cellular variants is provided in Supplementary Table S1, alongside with the selection strategy. The fact that subclones with stable dormant phenotype can be isolated from the aggressive parental cell line is something more than a technical opportunity, but might reveal something more profound about the biology of the dormant phenotype, i.e., that heritable characteristics of single cells, most likely epigenetically specified, are as important as the dormant microenvironment to dictate the choice between quiescence and proliferation.

The second option is the use of cell line series generated from a common precursor, but then selected independently from different animals (Supplementary Table S1). A notable example of these cell lines is the D2 series (D2.A1, D2.1, D2.0R) established by Fred Miller lab and characterized by Ann Chambers lab in her pioneering works on cancer dormancy (Mahoney et al., 1985; Aslakson and Miller, 1992; Rak et al., 1992; Morris et al., 1993, 1994). These cells have been cloned from spontaneously growing tumor in different BALB/cfC_3_H mice transplanted with a D2 hyperplastic alveolar nodule (HAN) line (Medina, 1976). D2.0R and D2.A1 cells grow with comparable rate on plastic, but with extremely different dynamics in 3D systems, coculture models and *in vivo*: A1 form overt metastases in lung and liver, OR lie dormant in the same organs for several months (Naumov et al., 2002; Barkan et al., 2008; Shibue and Weinberg, 2009; Touny et al., 2014; Montagner et al., 2020). Notably, another breast cancer cell series of great interest has been developed by the same laboratory in BALB/c mice. These cells show progressive acquisition of aggressive traits, from primary tumor growth, local invasion, intravasation, lung homing, overt metastasis (67NR > 168FARN > 4T07 > 4T1) (Aslakson and Miller, 1992). Often used in studies about dormancy, the cell line 4T07 was generated by sequential intravenous injection and isolation from lungs of a thioguanine- and ouabain-resistant cell line (Dexter et al., 1978; Blazar et al., 1980; Miller et al., 1987). The comparison between the two cell lines has led to the discovery of important molecules involved in the dormant state of lung, bone and brain disseminated cells (Gao et al., 2012, 2016).

A third option is the comparison among cell lines from completely different origin. Examples of these classes are the widely used triple negative cell line MDA-MB-231 (on the aggressive side of the spectrum) and the ER+ cells MCF7, T47D, ZR-75-1 that form quiescent metastatic lesions upon intravenous injection (Harrell et al., 2006; Holen et al., 2016; Wright et al., 2016; Gawrzak et al., 2018). Recently, bone metastatic versions of MCF7 cell line have been developed (Pavlovic et al., 2015; Clements and Johnson, 2019).

The last option is the comparison within the same cell line. This approach is a valuable alternative whenever the question is related to the drivers of cellular heterogeneity within the same population *in vitro* (Ghajar et al., 2013).

## Dormant Niche Components

**FIGURE 2:**
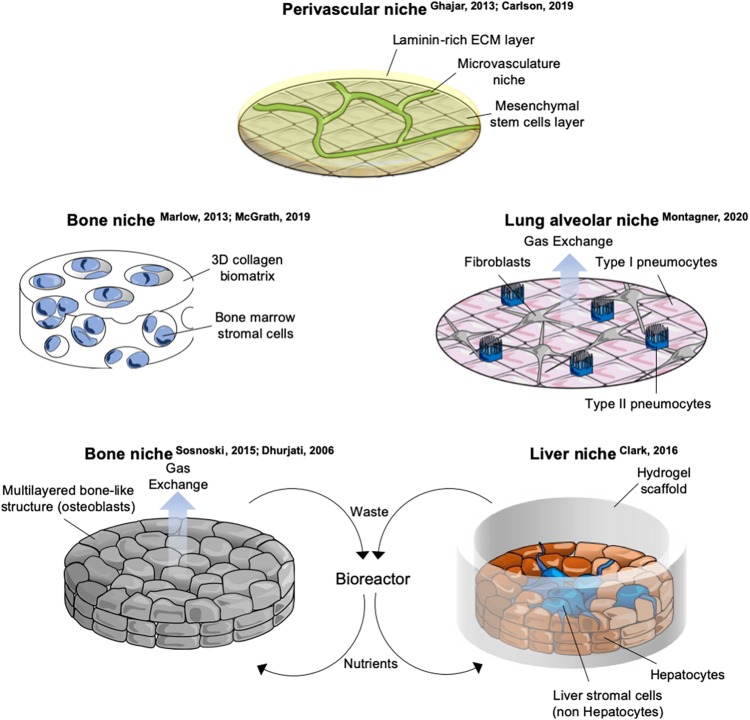
Schematics of selected *in vitro* organotypic systems designed for studying breast cancer cells (BCCs) metastatic dormancy. Schemes of *in vitro* systems developed to mimic microenvironments of specific metastatic organs. Details and references of each system are provided in Supplementary Table S2.

During the last two decades, the role of the microenvironment has been gaining importance in understanding several steps of the malignant transformation. For metastatic dormancy, the context where cells disseminate is key, as these cells are likely not to gain further mutations once they have entered quiescence. Components of the dormant niche include, but are not limited to: tissue architecture (geometry and stiffness, adhesion, cell density, ECM), biophysical (shear stress, tissue stiffness) and biochemical (oxygen levels, ROS concentration, nutrients, metabolites) environment, stromal populations. Examples and details of *in vitro* systems including tissue architecture and stromal cells is provided in Figure 2 and Supplementary Table S2.

### Tissue Architecture

The rapid development of bioengineering and a better understanding of the principles behind mechanotransduction (Iskratsch et al., 2014; Piccolo et al., 2014; Dupont, 2016; Montagner and Dupont, 2020) has led to several *in vitro* approaches to study metastatic dormancy of BCC. Models involving scaffolds of natural or artificial biomaterials, microfabrication, microfluidics, bioreactors, implantable niches have been developed (extensively reviewed in Pradhan et al., 2018; Rao et al., 2019). 3D spheroids can be generated simply as clusters of cells floating into medium (Wenzel et al., 2014; Cavnar et al., 2015; Imamura et al., 2015) or by employing natural (collagen-I, hyaluronic acid, and Cultrex) (Barkan et al., 2008; Fang et al., 2016; Kassim et al., 2017) or synthetic biomaterials (hydrogels of silica-polyethylene, polycaprolactone scaffolds) (Guiro et al., 2015; Preciado et al., 2017). Cells within these structures showed different degrees of quiescence, apoptosis, hypoxia and have been tested for their sensitivity to drugs. However, same caveats apply and, although informative, these models require more validation to address if their findings translate in a dormant phenotype. A notable exception is the well-known 3D system developed in Green laboratory. In this *in vitro* model, D2 cells lie on top of a stiff layer of basement membrane matrix and are embedded in a second layer of diluted basal matrix. Under these conditions D2.0R cells remain dormant in round structures, while D2.A1 continuously grow and invade surrounding territories. This conformation can then be further functionalized by adding other ECM proteins, such as collagen-I that drives the proliferative switch of otherwise dormant cells (Barkan et al., 2008, 2010). This system has been extensively validated *in vivo* and by other laboratories as well (Shibue and Weinberg, 2009; Shibue et al., 2012), and led to the discovery of the integrin-Src-ERK axis in the dormant-to-proliferation switch (Barkan et al., 2008, 2010). The use of ECM proteins can be combined with other niche components such as stromal cells to increase the complexity of the system (Ghajar et al., 2013).

### Stromal Cells

Resident organ parenchymal cells are an essential component of the dormant niche contributing to each step of quiescence-to-proliferative switch. Difficulties in the coculture of BCCs together with stromal cells are primarily two: (i) availability of organ-specific stromal cells and (ii) finding the culturing protocol that allows survival of all the cellular components. Moreover, the cellular composition of a tissue is often dynamic and heterogeneous, including different lineages of the same cell type as well as specific resident and transient immune populations. This complexity is often not captured by current *in vitro* models. Cocultures developed so far involving BCCs include osteoclasts (Lu et al., 2011), osteoblasts (Sosnoski et al., 2015), lung alveolar cells (Montagner et al., 2020), endothelial cells (Ghajar et al., 2013; Carlson et al., 2019), hepatocytes and non-hepatocytes liver stromal cells (Wheeler et al., 2014), bone marrow stromal cells (Ghajar et al., 2013; Marlow et al., 2013; Carpenter et al., 2018), neutrophils (Albrengues et al., 2018), peripheral blood mononuclear cells (Carpenter et al., 2018; Figure 2 and Supplementary Table S2). While for some populations primary cells are available [lung and bone marrow stromal cells (Ghajar et al., 2013; Marlow et al., 2013; Carpenter et al., 2018), hepatocytes and non-hepatocytes (Clark et al., 2016), NK cells (Malladi et al., 2016), neutrophils (Albrengues et al., 2018), human osteoblasts (Sosnoski et al., 2015), mouse SNO osteoblast-like cells (Capulli et al., 2019)], other cells require immortalization [endothelial cells (Ghajar et al., 2013), fibroblasts and type1-like pneumocytes (Montagner et al., 2020), human fetal osteoblasts and mesenchymal cells of bone marrow origin (Marlow et al., 2013), spontaneously immortalized mouse calvaria osteoblasts (Sosnoski et al., 2015)] or transformation to be cultivated [murine preosteoclasts (Lu et al., 2011), type2-like pneumocytes (Montagner et al., 2020)] and this might influence the correct crosstalk with the dormant BCC. Moreover, it has been shown that fibroblasts and endothelial cells have organ-specific gene expression (Chang et al., 2002; Nolan et al., 2013) and thus using unmatched stromal cells might overlook organ specific signaling. On the other side, the use of immortalized, homogeneous stromal cells allows a precise and repeatable experimental setup compared to deriving primary cells. An important detail in the *in vitro* models cited above is the use of a very low number of BCCs relative to stromal cells (Ghajar et al., 2013; Wheeler et al., 2014; Montagner et al., 2020).

### Biochemical Environment

Mitogens and nutrients directly impact on cell-cycle machinery (Pack et al., 2019), it is not surprising that decreasing their concentration in culture medium to a more physiological level already has an effect on proliferation. In a recently developed lung organotypic system, we used a Mitogen Low Nutrient Low medium (MLNL) that didn’t have a different effect on D2 cells *per se*, but that allowed to pinpoint some factors of the signaling network after stromal cells were added (Montagner et al., 2020). Mitogen Low Medium (MLM) alone had a remarkable effect on HCC1954-LCC1 (Latency Competent Cells) cells instead (Malladi et al., 2016). Cultivating LCC1 subclones in MLM medium drove expression of quiescence genes, such as Sox9, downregulation of several mediators of anti-tumor responses from NK cells and downregulation of Wnt, myc, NF-kB pathways, higher TGFβ response and lower P-ERK/P-p38 ratio (Malladi et al., 2016).

### Hypoxia

Oxygen concentration for most of the tissues oscillates between 5 and 7%, compared to the 20% in air at normal atmospheric pressure (McKeown, 2014). Bone marrow is a particularly hypoxic environment (Spencer et al., 2014) and a favorable metastatic site for BCCs. The use of physiological oxygen levels decreases proliferation for most of cells (Hubbi and Semenza, 2015) and, as with low serum, it might not be specific to dormant cells (de Prati et al., 2017; Lee et al., 2018). However, hypoxia has been implicated in dormancy in two studies where it has been shown to repress LIFR-STAT3 pathway leading to metastatic outgrowth (Johnson et al., 2016) and to preset primary tumor cells with a dormant program, then manifested after dissemination (Fluegen et al., 2017).

### Cell Density

Plating cells at a clonogenic density *in vitro* is already sufficient to induce heterogeneous growth arrest in BCCs. The Wieder laboratory developed an *in vitro* system of bone marrow dormancy that, despite its simplicity, has been shown to recall several aspects of quiescence validated in other laboratories. BCCs that are plated onto fibronectin-coated plates undergo quiescence in presence of FGF2 and activation of integrin α5β1, PI3K and ERK pathways. These cells express partial EMT markers and can re-enter proliferation upon treatment with IL6/8 and TGFβ) (Korah et al., 2004; Najmi et al., 2005; Barrios and Wieder, 2009; Tivari et al., 2015).

## Validation of *In Vitro* Models of Dormancy

What are we really modeling? This is the first question when designing any model and although this is an issue not unique to the topic of this review, the limited availability of clinical data makes it harder to unambiguously describe a dormant cell *in vitro*. Because an unequivocal list of dormant cells’ features is unavailable, several groups have validated their models by looking at a number of aspects that justified the parallel between the proposed *in vitro* model and the *in vivo* evidences, although a single model encompassing all of them has not yet been developed (Figure 1).

### Reversible Quiescence

The most important behavior underlying the dormant phenotype is growth arrest, and most of the *in vitro* models discussed in this review successfully achieve cell-cycle arrest of cells that can be reversed upon changing experimental conditions, such as serum levels, oxygen tension or with specific signals, such as inflammation. However, this does not demonstrate the relevance of the model. For example, it has been shown that adjusting the mechanical properties of the cell culture surface (using ECM-conjugated polyacrylamide gels) alone has a dramatic impact on cellular proliferation *in vitro* (Tilghman et al., 2012), but this does not imply that changes in local tissue mechanics are cause of entry and exit from dormancy. Ideally, the model conditions should be based on appropriate measurements of the *in vivo* environment in which dormant cells are found in terms of biophysical, biochemical and cellular composition of the niche (ECM composition and architecture, nutrients and metabolites concentration, ligands concentration, communication with stromal cells). However, this information is hard to determine at single-cell resolution in murine models and even harder to measure in clinical material. To distinguish between quiescence and senescence (or even apoptosis), cells must re-enter the proliferative state upon withdrawal of the factors used to trigger dormancy or upon treatment with signals able to drive exit from dormancy. Examples of such signals for BCCs are inflammation (LPS, smoke) (Cock et al., 2016; Albrengues et al., 2018), POSTN (Montagner et al., 2020), TGFβ1 (Ghajar et al., 2013), RTKs (Tivari et al., 2015; Montagner et al., 2020), IL6, Collagen I (Barkan et al., 2010), Src (Barkan et al., 2010; Montagner et al., 2020), SFRP2 (Montagner et al., 2020), IKKβ (Lamiaa et al., 2017), integrins activation (as discussed below); while examples of inhibitors are: TSP1 (Ghajar et al., 2013), p38 (Marlow et al., 2013), Alk5 (Marlow et al., 2013), BMP2 (Gao et al., 2012), TGFβ2 (Bragado et al., 2013), MSK1 (Gawrzak et al., 2018), IFN-β (Lan et al., 2019).

### Markers of Dormancy

Together with a reversible growth arrest, expression of gene/protein marker of dormancy should be addressed. Not many well-established markers are available for BCCs, those that have been widely validated *in vitro* and *in vivo* so far include DEC2/SHARP1, p27, NR2F1 and the ratio between P-ERK/P-p38 proteins (Touny et al., 2014; Johnson et al., 2016; Linde et al., 2016; Malladi et al., 2016; Borgen et al., 2018). We recently reported an RNA-seq analysis of lung-disseminated dormant BCCs that will hopefully provide new markers for the characterization of these cells *in vitro* (Montagner et al., 2020).

Regardless of the metrics adopted, the predictive power of an *in vitro* system represents its best validation and testing the predictions generated in mice or patients is the ultimately goal (Figure 1).

## Organ-Shared Mechanisms of Dormancy

Whether the same mechanisms for quiescence or reawakening are shared among different organs *in vivo* is unknown. The observations that dormant subclones isolated from one organ show quiescence in other organs suggests that there might be some overlap and thus either intrinsic genetic/epigenetic mechanisms dominate over microenvironmental cues or there are common traits in very different niches. For example, D2.0R cells are dormant in liver and lung, HCC1954-LCC1 are derived from brain disseminated cells, but are found latent in lungs as well (Malladi et al., 2016), T47D-DBM have been isolated as bone dormant variant (Gawrzak et al., 2018), but they survive in quiescent state in lungs as well (Montagner et al., 2020). Mechanistic similarities between dormancy in different organs will aid the development of universal clinical strategies with the ability to eliminate dormant cells regardless of their anatomical site.

Activation of ERK and p38, associated with metastatic outgrowth and quiescence, respectively, have been consistently observed in bone and lungs (Barkan et al., 2010; Touny et al., 2014; Linde et al., 2016; Malladi et al., 2016; Gawrzak et al., 2018). THBS1 from PVN (perivascular niche) was shown to induce dormancy in lung and bone marrow (Ghajar et al., 2013). Src has been validated as BCCs survival signal in bone and lungs (Barkan et al., 2010; Zhang et al., 2013; Montagner et al., 2020). Similarly, Akt was found as a factor supporting survival or outgrowth of BCCs in bone *in vivo* (Zhang et al., 2013) and *in vitro* (Korah et al., 2004) and in an *in vitro* model of lung (Montagner et al., 2020). The proinflammatory cytokine LPS induces outgrowth of quiescent cells in lung (Cock et al., 2016; Albrengues et al., 2018) and in an *in vitro* model of dormancy in the liver (Clark et al., 2018).

A recurring theme in several models of BCCs dormancy is the importance of integrins into survival or chemoresistance. Many groups independently reported the key role of different integrin dimers. Integrinβ_1_-dependent activation of Src and ERK downstream of collagen-I has been found to drive exit from quiescence *in vitro* and *in vivo* (Barkan et al., 2010; Touny et al., 2014), to sustain reawakening of dormant cells following NET proteolysis of laminin *in vivo* [in the α_3_β_1_ form (Albrengues et al., 2018)] and to support survival after engagement of fibronectin in *in vitro* bone dormancy models [in the α_5_β_1_ form (Barrios and Wieder, 2009; Barney et al., 2019)]. Perivascular-driven chemoresistance of dormant BCCs also relies on α_5_β_3_ and α_4_β_1_ activation by von Willebrand Factor and VCAM-1 in endothelium, respectively. By using blocking antibodies against those isoforms in combination with doxorubicin and cyclophosphamide Carlson et al. (2019) were able to circumvent chemoresistance and decrease tumor burden in bone marrow. Finally, we recently found that acute treatment of mice with cilengitide (inhibitor of α_v_β_3_, α_v_β_5_ and α_5_β_1_ integrins) effectively reduced lung-disseminated dormant BCCs (Montagner et al., 2020). In sum, quiescent BCCs seem to rely on integrins in many ways and might prove more sensitive to integrin inhibitors then established or actively growing cancer cells.

## Concluding Remarks

So far, the battle to defeat metastatic breast cancer has achieved only limited advances since the advent of hormone target therapies. For some types of cancer, the period metastatic dormancy offers an opportunity to eliminate disease before it resumes aggressive growth, but the inherent lack of data from patients slows down the development of new therapies. The development of *in vitro* models to bypass this limitation has been the goal for several laboratories during the last decade, and common themes in the survival and growth of disseminated BCCs in different organs are starting to emerge. Here we present cellular models and microenvironmental factors implemented so far, together with a critical discussion on validation strategies. The discovery of new markers from patients and validation of same mechanisms among different systems will give confidence to translate these findings into clinical trials and hope to finally impact on the origin and development of metastatic breast cancer.

## Author Contributions

MM and ES equally contributed to conceiving and writing the review.

## Conflict of Interest

The authors declare that the research was conducted in the absence of any commercial or financial relationships that could be construed as a potential conflict of interest.
