# Supplementary material for: In vitro Models of Breast Cancer Metastatic Dormancy
Source: Front Cell Dev Biol. 2020 Mar 3;8:37. doi: 10.3389/fcell.2020.00037 (PMC7062644; doi:10.3389/fcell.2020.00037)
Supplement: Supplementary file 1 [file Table_1.pdf]

Supplementary Table 1. *In vivo* dormant breast cancer cell lines.

Cell line	ER status. subtype	Known *in vivo* tropism	Origin	Subtype	Refs. for *in vivo* and *in vitro* use
MCF-7	ER^+/PR^+	Lung^{A,D} (Albrengues et al., 2018; Harrell et al., 2006). Moderately metastatic: bone^{B,D} (Carlson et al., 2019; Holen et al., 2016; Wright et al., 2016)	Human, Met pleural effusion	LumA	Albrengues et al., 2018; Barkan et al., 2008; Barney et al., 2019; Barrios & Wieder, 2009; Carlson et al., 2019; Ghajar et al., 2013; Holen et al., 2016; Imamura et al., 2015; Korah et al., 2004; Lee et al., 2018; Marlow et al., 2013; Najmi et al., 2005; Sowder & Johnson, 2018; Wheeler et al., 2014; Wright et al., 2016
ZR-75-1	ER^+/PR^+	Bone^{B,D} (Gawrzak et al., 2018; Wright et al., 2016)	Human, Met ascites	LumA	Marlow et al., 2013; Wright et al., 2016; Fluegen et al., 2017; Gawrzak et al., 2018; Barney et al., 2019
T-47D	ER^+/PR^+	LN, lung^{D} (Harrell et al., 2006), bone^{D} (Holen et al., 2016)	Human, Met pleural effusion	LumA	Korah et al., 2004; Najmi et al., 2005; Harrell et al., 2006; Marlow et al., 2013
4T07	Un.	LN, lung [aggressive^{A} (Miller et al., 1987), dormant^{C}, Gao et al., 2012), bone marrow^{C}, Carlson et al., 2019]	*In vivo* and *in vitro* selection of a spontaneous mammary tumour from BALB/cfC3H mice	Un.	Aslakson and Miller, 1992; Gao et al., 2012, 2016; Malladi et al., 2016; Carlson et al., 2019
D2.0R	ER^+/PR^+	Lung^{A} (Albrengues et al., 2018; Barkan et al., 2010), liver^{C,E} (Naumov et al., 2002)	Primary tumor from D2 preneoplastic mammary hyperplastic alveolar nodule line	LumA	Naumov et al., 2002; Barkan et al., 2008, 2010; Shibue and Weinberg, 2009; Touny et al., 2014; Albrengues et al., 2018; Sowder and Johnson, 2018
HCC1954-LCC1	ER^-/PR^- (parental line)	Brain^{B} (Malladi et al., 2016), lung (Malladi et al., 2016)	Recovered from metastasis-free lungs of mice injected with HCC1954 cells	Her2 (parental HCC1954)	Malladi et al., 2016
D2.A1-d	ER^-/PR^- (parental line)	Lung^{A} (Cock et al., 2016)	Ten rounds of isolation from lungs of mice injected in tail vein	LumB (parental D2.A1)	Cock et al., 2016
MDA-MB-231-SCP6	ER^-/PR^- (parental line)	Bone^{B} (Lu et al., 2011)	MDA-MB-231 subclone with low expression of bone-metastasis signature	TNBC (parental MDA-MB-231)	Kang et al., 2003; Lu et al., 2011
HMT-3522-T4-2	ER^-/PR^- (parental line)	Weakly metastatic: lung^{B} (Ghajar et al., 2013), bone marrow^{B} (Ghajar et al., 2013), brain^{B} (Ghajar et al., 2013)	*In vitro* and *in vivo* selection of a weakly tumorigenic cell line derived from a fibrocystic lesion	TNBC (parental HMT-3522)	Briand and Lykkesfeldt, 2001; Ghajar et al., 2013
T47D-DBM	ER^+/PR^+ (parental line)	Bone^{B} (Gawrzak et al., 2018)	*In vivo* selection from metastasis-free bones of mice injected with T47D cells	LumA (parental T47D)	Gawrzak et al., 2018

A: after tail vein injection
B: after intracardiac injection
C: from primary tumor
D: from primary tumor with Matrigel or Cultrex
E: from mesenteric vein injection
Un.: Unknown
