# Supplementary material for: In vitro Models of Breast Cancer Metastatic Dormancy
Source: Front Cell Dev Biol. 2020 Mar 3;8:37. doi: 10.3389/fcell.2020.00037 (PMC7062644; doi:10.3389/fcell.2020.00037)
Supplement: Supplementary file 2 [file Table_2.pdf]

Supplementary Table 2. Organotypic *in vitro* systems for the study of metastatic dormancy.

Description	Stromal cells	Breast cancer cells tested	Validation strategies	Refs.
Bone				
3D-collagen biomatrix seeded with human bone marrow stromal cells. Matrixes with defined stromal composition provided reversible growth arrest signals *in vitro* and upon implantation *in vivo* (inhibitory niche). Specific inhibition of RTK, Alk5, and p38 signaling in the inhibitory niche results in increased proliferation of BCCs without affecting stromal cells.	Supportive niche:<br>Primary human mesenchymal stem cells<br>Inhibitory niche:<br>hFOBs (human foetal osteoblasts)<br>HS-5 (human mesenchymal cells of bone marrow origin)<br>HUVEC (human umbilical cord endothelial cells)	SUM149, SUM159, MDA-MB-231, BT474, MCF7, T47D, ZR75-1	Reversible growth arrest, Ki67 staining, cell cycle arrest markers (p21, p27), response to signals	Marlow et al., 2013; McGrath et al., 2019
BCCs are added together with laminin-rich ECM to human microvasculature niche. Within this system, BCCs show heterogeneous behavior, with cells adjacent to mature capillaries remaining dormant (via TSP-1), and cells growing closer to neovascular tips due to expression of POSTN and TGFβ1. This *in vitro* model has been further utilized to dissect signals that sustain chemoresistance of dormant BCCs *in vivo*.	HUVEC (human umbilical cord endothelial cells)<br>Primary human mesenchymal stem cells	HMT-3522-T4-2, MCF7, MDA-MB-231	Reversible growth arrest, Ki67 staining, cell cycle arrest markers, response to signals	Ghajar et al., 2013; Carlson et al., 2019
Osteoblasts can be cultivated for several weeks in a bioreactor forming a multilayered bone-like structure as substrate for BCCs. Bone remodeling cytokines have been shown to drive proliferation of quiescent BCCs in this organotypic model.	MC3T3-E1 (mouse calvariaosteoblasts)<br>NHOst (human osteoblasts)<br>hFOBs (human foetal osteoblasts)	MDA-MB-231 BRMS1, MCF7	Reversible growth arrest, Ki67, response to signals	Dhurjati et al., 2006; Sosnoski et al., 2015
Liver				
Polystyrene (or hydrogel) scaffold and coated with collagen I, then primary hepatocytes and non-hepatocytes liver stromal cells are added and allowed to form the organ-like system. The system has been validated by testing proliferative signals (such as LPS) and exploited to uncover potential biomarkers released from the stroma in response to disseminated BCCs.	Primary human hepatocytes and non-hepatocytes	MDA-MB-231, MCF7	Reversible growth arrest, Ki67/EdU, cell shape, response to signals	Wheeler et al., 2014; Clark et al., 2016, 2018
Lung				
Alveolar type1-like cells, alveolar type2-like cells and lung fibroblasts are cultured with mitogen and nutrient low medium on an air-permeable surface to recreate the gas flow through the alveolar wall.	TT1 (human type-1 pneumocytes)<br>H441 cells (human lung adenocarcinoma type-2 pneumocytes)<br>Human lung fibroblasts	D2.OR, D2.A1, MCF7, T47D-DBM, 4T07	Reversible growth arrest, Ki67 staining, cell shape, gene expression, response to signals	Montagner et al., 2020
